# *SPATA5* mutations cause a distinct autosomal recessive phenotype of intellectual disability, hypotonia and hearing loss

**DOI:** 10.1186/s13023-016-0509-9

**Published:** 2016-09-29

**Authors:** Rebecca Buchert, Addie I. Nesbitt, Hasan Tawamie, Ian D. Krantz, Livija Medne, Ingo Helbig, Dena R. Matalon, André Reis, Avni Santani, Heinrich Sticht, Rami Abou Jamra

**Affiliations:** 1 Institute of Human Genetics, Friedrich-Alexander-University Erlangen-Nuremberg, 91054 Erlangen, Germany; 2 Institute of Medical Genetics and Applied Genomics, University of Tübingen, 72076 Tübingen, Germany; 3 Division of Genomic Diagnostics, Children’s Hospital of Philadelphia, Philadelphia, PA 19104 USA; 4 Division of Human Genetics, The Children’s Hospital of Philadelphia and the Perelman School of Medicine at the University of Pennsylvania, Philadelphia, PA 19104 USA; 5 Division of Child Neurology, Departments of Pediatrics and Neurology, Children’s Hospital of Philadelphia and the Perelman School of Medicine at the University of Pennsylvania, Philadelphia, PA 19104 USA; 6 Division of Neurology, The Children’s Hospital of Philadelphia, 34th St. and Civic Center Blvd., Philadelphia, PA 19104-4399 USA; 7 Department of Neuropediatrics, University Medical Center Schleswig-Holstein, Kiel Campus, Kiel, Germany; 8 Department of Pathology & Laboratory Medicine, Perelman School of Medicine at the University of Pennsylvania, Philadelphia, PA 19104 USA; 9 Institute of Biochemistry, Friedrich-Alexander-University Erlangen-Nuremberg, 91054 Erlangen, Germany; 10 Institute of Human Genetics, University of Leipzig Hospitals and Clinics, 04103 Leipzig, Germany

**Keywords:** ARID, Microcephaly, Hearing loss, Hypotonia, NGS

## Abstract

We examined an extended, consanguineous family with seven individuals with severe intellectual disability and microcephaly. Further symptoms were hearing loss, vision impairment, gastrointestinal disturbances, and slow and asymmetric waves in the EEG. Linkage analysis followed by exome sequencing revealed a homozygous variant in *SPATA5* (c.1822_1824del; p.Asp608del), which segregates with the phenotype in the family. Molecular modelling suggested a deleterious effect of the identified alterations on the protein function. In an unrelated family, we identified compound heterozygous variants in *SPATA5* (c.[2081G > A];[989_991delCAA]; p.[Gly694Glu];[.Thr330del]) in a further individual with global developmental delay, infantile spasms, profound dystonia, and sensorineural hearing loss. Molecular modelling suggested an impairment of protein function in the presence of both variants.

SPATA5 is a member of the ATPase associated with diverse activities (AAA) protein family and was very recently reported in one publication to be mutated in individuals with intellectual disability, epilepsy and hearing loss. Our results describe new, probably pathogenic variants in *SPATA5* that were identified in individuals with a comparable phenotype. We thus independently confirm that bi-allelic pathogenic variants in *SPATA5* cause a syndromic form of intellectual disability, and we delineate its clinical presentation.

**Electronic supplementary material:**

The online version of this article (doi:10.1186/s13023-016-0509-9) contains supplementary material, which is available to authorized users.

## Main text

Intellectual disability is characterized by significant limitations in both intellectual functioning and in adaptive behavior and has a prevalence of about 2 % of the population [1]. The cause for intellectual disability is often genetic and therefore, it is one of the most common reasons for genetic counselling and poses a major socio-economic burden worldwide. Recent studies have shown that in many cases point mutations, which occur *de novo* or are inherited through autosomal recessive or X-linked traits, are responsible for the phenotype [2, 3]. Very recently, several affected individuals with various variants in *SPATA5* (MIM 616577) have been reported [4, 5]. In our cohort of about 200 families with intellectual disability, we identified a homozygous deletion of one amino acid in *SPATA5* in a consanguineous family with seven affected members. In an unrelated affected individual with sporadic global developmental delay and epileptic encephalopathy we identified compound heterozygous variants in *SPATA5* in the context of clinical exome sequencing.

This study was approved by the Ethics Committees of the University Bonn and the University Erlangen-Nuremberg in Germany and informed consent of all examined persons or of their guardians recruited in the course of this study was obtained. Inclusion of a single individual followed at the Children’s Hospital of Philadelphia, USA was IRB-exempt.

**Fig. 1:**
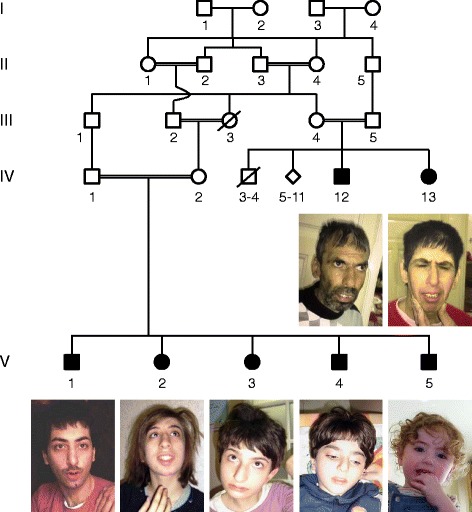
Pedigree and pictures of family MR003

Family MR003 is a large Syrian family with multiple consanguineous marriages and seven affected individuals (Fig. 1). Affected individuals IV-12 and IV-13 are uncle and aunt of the affected individuals V-1 to V-5. Pregnancy, delivery, and perinatal period were reported to be uneventful for all individuals of family MR003 except for individuals IV-13, V-3, and V-4. Individual IV-13 was born with cyanosis and neonatal hypotonia; individuals V-3 and V-4 had neonatal hypotonia after an unremarkable delivery. All affected individuals reached developmental mile stones including gross motor milestones such as unsupported sitting and walking at a late normal range. Affected individuals were examined at the age of 41 years (IV-12), 39 years (IV-13), 22 years (V-1), 20 years (V-2), 12 years (V-3), 5 years (V-2) and 2 years (V-5). Common symptoms of all affected members are moderate to severe intellectual disability, severely limited receptive and expressive speech, and microcephaly (Additional file 1: Table S1). Seizures were not reported in either of the family members. EEG in V-1 showed slow and asymmetric fronto-central waves. Individuals IV-12, IV-13, and V-2 presented with hearing impairment as determined by Auditory Evoked Potential, other affected individuals were not tested, thus, we cannot exclude mild hearing impairment in these individuals. Individuals IV-12 and IV-13 presented with vision impairment especially at night. Gastrointestinal disturbances, such as constipation, are observed in individuals IV-12 and IV-13. All affected individuals demonstrate autistic features including reduced eye contact. Additionally, V-2 shows stereotypic movements and V-1 has some degree of auto-aggression and is easily frightened. Individual V-3 presented with central muscular hypotonia and individuals IV-12 and IV-13 have a decreased ability to ambulate, like through a combination of central muscular hypotonia and decreased coordination. All affected individuals have a normal sleep pattern. Facial features include a long nose with underdeveloped alae nasi and arched eyebrows. Apart from presenting with microcephaly, growth parameters were in the normal range for all individuals.

Metabolic work-up of individual V-2 showed normal amino acids and urinary organic acids. Karyotype and ocular fundus was unremarkable. Brain MRI for IV-13 at an adult age indicated brain atrophy. Brain MRIs for individuals V-1 and V-2 at ages of 2 and 4 years, respectively, were unremarkable.

**Fig. 2:**
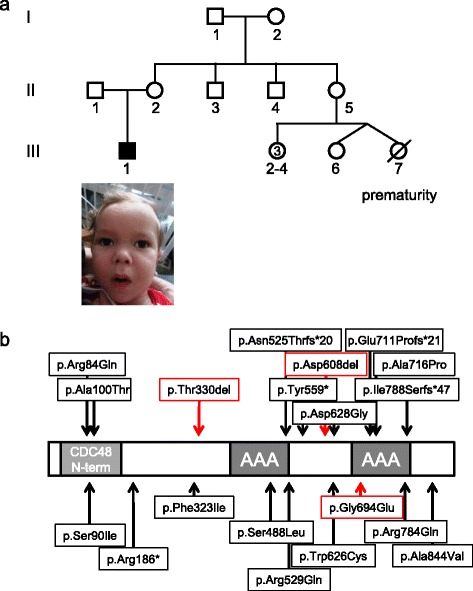
**a** Pedigree and picture of family B. **b** Schematic structure of SPATA5 and identified alterations. Previously reported variants are indicated in *black*, the variants identified in this study c.1822_1824del; p.Asp608del, c.2081G > A; p.Gly694Glu and c.989_991delCAA; p.Thr330del are indicated in *red*

In family B, an outbred family of European ancestry, only one child, B1, is affected (Fig. 2a). The proband was born after a pregnancy complicated by maternal diabetes mellitus. Delivery was uneventful at 41 weeks of gestation. The affected individual was hypotonic and profoundly delayed with failure to achieve any major developmental milestones. He was referred to early intervention for developmental delay at a young age. He failed initial brainstem auditory evoked responses (BAERS) as a neonate. A hearing test was repeated later on and he was diagnosed with sensorineural hearing loss. At 8 months he had a percutaneous gastrostomy tube placed for failure to thrive. The proband started having infantile spasms at the age of 8 months. A head CT and brain MRI were unremarkable. A video EEG showed hypsarrythmia. He was treated with Lacosamide and Levetiracetam. At 17 months he was admitted to the Children’s Hospital of Philadelphia for break-through seizures in the setting of pneumonia, but has remained seizure-free with an unremarkable follow-up EEG. Ophthalmologic examination revealed poor vision likely secondary to cortical blindness. Cardiology evaluation noted a fenestrated atrial septal defect. The proband had a movement disorder, which developed around 10 months of age, characterized by profound dystonic posturing of upper limbs greater than lower limbs with neck involvement in the absence of spasticity. At 17 months, the proband was unable to sit independently or roll over. He was able to hold objects intermittently but unable to transfer them. He did not smile or coo. His growth parameters were in the low normal range (weight <5th percentile, length 9th percentile and head circumference 5th percentile). He had divergent strabismus, low nasal bridge and broad eyebrows, roving eye movements, scoliosis, and a sacral tuft of hair.

Linkage analysis for family MR003 was performed using the genotype data of Human610-Quad DNA Analysis BeadChips (Illumina, San Diego, CA, USA) and resulted in the identification of single candidate region on chromosome 4:122,982,313-133,038,675 bp, with a size of 10 Mb as previously published [6]. Whole-exome sequencing (WES) was performed on an Illumina Genome Analyzer IIx (Illumina, San Diego, CA, USA) after enrichment with the SureSelect Human All Exon Kit (Agilent, Santa Clara, Ca, USA) as described previously [7]. Data was filtered for rare variants that were annotated with a minor allele frequency of ≤0.01 in the databases of 1000 Genomes [8] and Exome Variant Server (EVS), and that are within the candidate region. Then variants were prioritized according to predicted effect on protein sequence, and according to function of the encoded protein. Variants were validated and tested for segregation using Sanger sequencing. We identified a single homozygous candidate variant in *SPATA5* (NM_145207): c.1822_1824del; p.Asp608del that was predicted as damaging by *in silico* prediction tools.

Genomic DNA of affected individual B1 was extracted from blood with the AutoGen DNA extraction kit following manufacturer’s guidelines (AutoGen, Holliston, MA, USA). The WES library was prepared using the SureSelectXT Human All Exon V5 kit following standard manufacturer protocol (Agilent Technologies, Santa Clara, CA, USA) and sequenced on the Illumina HiSeq 2500 using the 2 × 100 bp kit, with the targeted average coverage of 150x in the proband and 100x in the mother (Illumina, San Diego, CA, USA). Alignment and variant calling were performed with an in-house bioinformatics pipeline. Variants with a minor allele frequency of <0.005 in the Exome Aggregation Consortium database and expected to affect coding/splicing of the protein, or were present in the Human Gene Mutation Database (HGMD) [9] were included in the analysis using the Bench Lab NGS software (Cartagenia, Cambridge, MA, USA). A pair of variants in *SPATA5*: c.989_991delCAA; p.Thr330del and c.2081G > A; p.Gly694Glu was identified based on their compound heterozygous inheritance. The proband’s mother was heterozygous for the p.Thr330 deletion. The p.Gly694Glu variant was not present in the mother; it is presumably inherited from the father who was not available. The c.989_991delCAA; p.Thr330del variant was previously described in a proband with overlapping phenotype [4].

To determine the effect of the identified alterations on the phenotype the amino acid sequence of SPATA5 was analysed using PFAM [10] and two AAA ATPase domains spanning residues 390–523 and 664–796, respectively, were identified. Then, modeling was performed with Modeller 9.9 [11] using the crystal structure of the hexamieric ATPase p97 (PDB code: 3CF2) [12] as a template. The resulting model was verified using PROCHECK [13] and WHATCHECK [14] and revealed a good stereochemistry and no steric clashes. The deletions p.Asp608del and p.Thr330del were created with ModLoop [15, 16]. RasMol [17] was used for structure analysis and visualization.

**Fig. 3:**
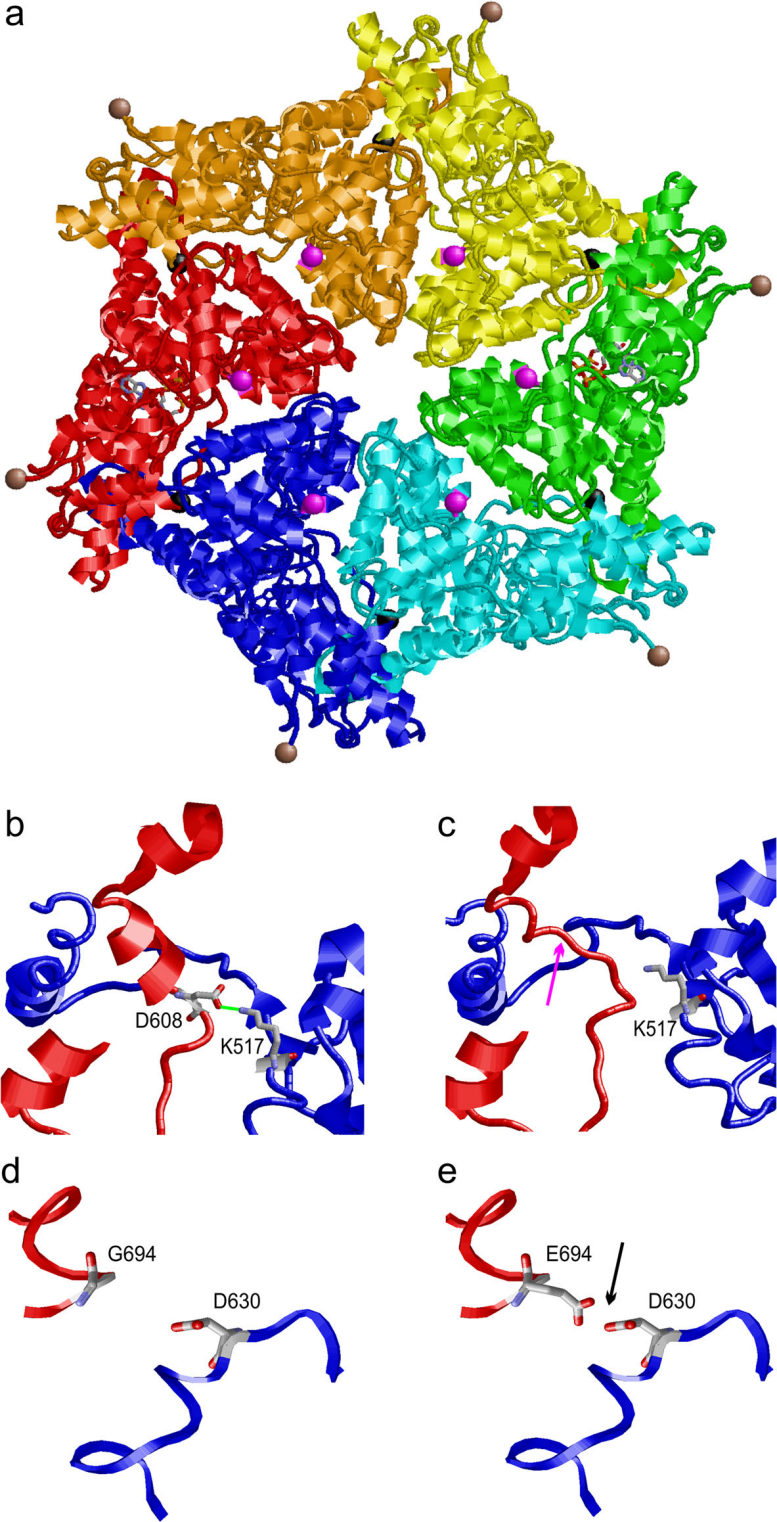
Structure of the AAA ATPase domains of SPATA5. **a** Model of the hexameric quaternary structure of SPATA5 showing the individual subunits in different colours. ADP-molecules bound to the ATPase domains are shown in stick presentation and coloured according to their atom type. p.Asp608 and p.Gly608Glu are located at the subunit interface and are depicted as black and magenta balls, respectively. pThr330 is located N-terminally adjacent to the globular domain and is shown as brown ball. **b** Enlargement showing the stabilizing interactions of p.Asp608 (D608) with p.Lys517 (K517) of the adjacent subunit (both residues are shown in stick presentation). The salt-bridge between both residues is shown in *green* and the subunits are coloured in *red* and *blue* respectively. **c** Deletion of p.Asp608 (D608) results in a loss of the helical secondary structure (*pink arrow*) and of the intersubunit salt-bridge. **d** Enlargement showing the location of pGly694 (G694) at the subunit interface. **e** Replacement of p.Gly544 by glutamate (E694) results in electrostatic repulsion (*black arrow*) with p.Asp630 (D630) of the adjacent subunit

The SPATA5 model (Fig. 3a) reveals that p.Asp608 is located at the subunit interface and forms stabilizing interactions with p.Lys517 of the adjacent subunit (Fig. 3b). Deletion of p.Asp608 causes a loss of the salt-bridge with p.Lys517 (Fig. 3c). In addition, the shorter loop in the mutant causes an unfolding of the α-helix, which is present N-terminally adjacent to the site of variant in the wildtype (Fig. 3b, c). These effects are expected to reduce the stability of both the hexamer and also of the individual subunits consequently leading to reduced enzymatic activity.

Similar to p.Asp608, p.Gly694 is also located at the subunit of the interfaces in the hexameric enzyme (Fig. 3d). A replacement of p.Gly694 by glutamate causes electrostatic repulsion with p.Asp630 of the adjacent protein subunit (Fig. 3e). Therefore, this alteration is expected to decrease hexamer stability in a similar fashion as p.Asp608del. However, the effect on the fold of the individual subunits appears less severe than for p.Asp608del.

Due to lack of sequence similarity to known proteins, the sequence stretch around p.Thr330 cannot be modelled in atomic detail. The location p.Thr330 in the N-terminal proximity of the globular AAA ATPase domain suggests that a deletion of this residue might still affect protein function and stability, although to a lesser extent compared to the p.Asp608del or p.Gly694Glu variants. The p.Thr330del variant was previously described as pathogenic [4].

*SPATA5* encodes a protein of 892 amino acids, which is part of the ATPase associated with diverse activities (AAA) protein family. Proteins of this family are characterized by their ATPase domain, which has a length of 200–250 amino acids and is highly conserved. AAA proteins usually form hexamer or heptamer rings with a central pore [18]. By hydrolysing ATP they can generate mechanical force, which is used for conformational remodelling of proteins or polynucleotides. Thus, members of the AAA protein family are thought to play an important role in protein degradation, DNA replication, membrane fusion events and in the movement of microtubules in various cellular compartments [18].

Variants in *LONP1*, a member of the closely related AAA+ family, have been reported to be causative for CODAS syndrome (MIM 600373). CODAS syndrome is characterized by intellectual disability, muscular hypotonia, epilepsy, sensorineural hearing loss, short stature, and skeletal abnormalities [19]. In addition, mutations in genes encoding for members of the AAA protein family have been reported in additional disorders including Zellweger syndrome (MIM 214100, *PEX1*), hereditary spastic paraplegia type 7 (MIM 607259, *SPAST*, *SPG7*), early-onset torsion dystonia (MIM 605204, *TOR1A*), and Paget disease with frontotemporal dementia (MIM 601023, *VCP*) [20–25]. Although these disorders differ significantly in their clinical presentation, neurological features are the major phenotype in all these disorders.

Very recently, several individuals with variants in *SPATA5* presenting with symptoms of microcephaly, intellectual disability, seizures and hearing loss were reported [4, 5]. All variants in *SPATA5* reported to date are located in important regions with an emphasis on the CDC48 N-terminal domain, the AAA domains or the subunit interface (Fig. 2b). Two of the variants reported here (p.Asp608del and p.Gly694Glu) are located at the subunit interface along with three previously reported variants and our modelling showed an impact on subunit oligomerization. The other variant reported here (p.Thr330del) was previously reported in individuals with a similar phenotype [4].

We conclude that the variants we identified in *SPATA5* are causative for the phenotype in the affected individuals. Our findings suggest that bi-allelic pathogenic variants in *SPATA5* may cause a phenotype with some distinct features. All individuals present with intellectual disability and gross motor delay out of proportion to the degree of intellectual impairment. Other neurological features can be variable. While the affected individual B1 presented with a profound movement disorder and epileptic encephalopathy characterized by infantile spasms with hypsarrhythmia, none of the seven affected individuals in family MR003 had seizures or dystonia. For many previously reported individuals movement disorders such as hypotonia, dystonia or spasticity have been described, as well. Most individuals have microcephaly, sensorineural hearing loss and cortical vision impairment. Autistic features were common in examined individuals. Neuroimaging shows non-specific abnormalities such as diffuse or general atrophy or a thin corpus callosum in some individuals while other individuals have a normal MRI. Many individuals also present with gastrointestinal issues. We suggest that pathogenic *SPATA5* variants can be identified in individuals with complex neurodevelopmental phenotypes with obligatory intellectual disability, particularly in individuals with concomitant hearing and vision impairment. Our report expands the known features of *SPATA5* encephalopathy and suggests that some of the clinical features, such as the presence or absence of epilepsy, may be mutation specific. We expect that further cases will add to the delineation of the SPATA5 phenotype.

## Additional file

Additional file 1: Table S1. Comparison of phenotypes of individuals with mutations in *SPATA5*. (DOCX 15 kb)

## Abbreviations

AAA: ATPase associated with diverse activities
ARID: Autosomal recessive intellectual disability
BAERS: Brainstem auditory evoked responses
EEG: Electroencephalography
HGMD: Human gene mutation database
WES: Whole exome sequencing

## References

[1] Leonard H, Wen X (2002). The epidemiology of mental retardation: challenges and opportunities in the new millennium. Ment Retard Dev Disabil Res Rev.

[2] Najmabadi H, Hu H, Garshasbi M, Zemojtel T, Abedini SS, Chen W, Hosseini M, Behjati F, Haas S, Jamali P (2011). Deep sequencing reveals 50 novel genes for recessive cognitive disorders. Nature.

[3] Rauch A, Wieczorek D, Graf E, Wieland T, Endele S, Schwarzmayr T, Albrecht B, Bartholdi D, Beygo J, Di Donato N (2012). Range of genetic mutations associated with severe non-syndromic sporadic intellectual disability: an exome sequencing study. Lancet.

[4] Tanaka AJ, Cho MT, Millan F, Juusola J, Retterer K, Joshi C, Niyazov D, Garnica A, Gratz E, Deardorff M (2015). Mutations in SPATA5 Are Associated with Microcephaly, Intellectual Disability, Seizures, and Hearing Loss. Am J Hum Genet.

[5] Kurata H, Terashima H, Nakashima M, Okazaki T, Matsumura W, Ohno K, Saito Y, Maegaki Y, Kubota M, Nanba E, et al. Characterization of SPATA5-related encephalopathy in early childhood. Clin Genet 2016. doi: 10.1111/cge.12813 10.1111/cge.12813 27246907

[6] Abou Jamra R, Wohlfart S, Zweier M, Uebe S, Priebe L, Ekici A, Giesebrecht S, Abboud A, Al Khateeb MA, Fakher M (2011). Homozygosity mapping in 64 Syrian consanguineous families with non-specific intellectual disability reveals 11 novel loci and high heterogeneity. Eur J Hum Genet.

[7] Abou Jamra R, Philippe O, Raas-Rothschild A, Eck SH, Graf E, Buchert R, Borck G, Ekici A, Brockschmidt FF, Nothen MM (2011). Adaptor protein complex 4 deficiency causes severe autosomal-recessive intellectual disability, progressive spastic paraplegia, shy character, and short stature. Am J Hum Genet.

[8] Abecasis GR, Auton A, Brooks LD, DePristo MA, Durbin RM, Handsaker RE, Kang HM, Marth GT, McVean GA, Genomes Project C (2012). An integrated map of genetic variation from 1,092 human genomes. Nature.

[9] Stenson PD, Mort M, Ball EV, Shaw K, Phillips A, Cooper DN (2014). The Human Gene Mutation Database: building a comprehensive mutation repository for clinical and molecular genetics, diagnostic testing and personalized genomic medicine. Hum Genet.

[10] Finn RD, Bateman A, Clements J, Coggill P, Eberhardt RY, Eddy SR, Heger A, Hetherington K, Holm L, Mistry J (2014). Pfam: the protein families database. Nucleic Acids Res.

[11] Webb B, Sali A (2014). Protein structure modeling with MODELLER. Methods Mol Biol.

[12] Davies JM, Brunger AT, Weis WI (2008). Improved structures of full-length p97, an AAA ATPase: implications for mechanisms of nucleotide-dependent conformational change. Structure.

[13] Laskowski RA, Rullmannn JA, MacArthur MW, Kaptein R, Thornton JM (1996). AQUA and PROCHECK-NMR: programs for checking the quality of protein structures solved by NMR. J Biomol NMR.

[14] Hooft RW, Vriend G, Sander C, Abola EE (1996). Errors in protein structures. Nature.

[15] Fiser A, Do RK, Sali A (2000). Modeling of loops in protein structures. Protein Sci.

[16] Fiser A, Sali A (2003). ModLoop: automated modeling of loops in protein structures. Bioinformatics.

[17] Sayle RA, Milner-White EJ (1995). RASMOL: biomolecular graphics for all. Trends Biochem Sci.

[18] Bar-Nun S, Glickman MH (2012). Proteasomal AAA-ATPases: structure and function. Biochim Biophys Acta.

[19] Strauss KA, Jinks RN, Puffenberger EG, Venkatesh S, Singh K, Cheng I, Mikita N, Thilagavathi J, Lee J, Sarafianos S (2015). CODAS syndrome is associated with mutations of LONP1, encoding mitochondrial AAA+ Lon protease. Am J Hum Genet.

[20] Watts GD, Wymer J, Kovach MJ, Mehta SG, Mumm S, Darvish D, Pestronk A, Whyte MP, Kimonis VE (2004). Inclusion body myopathy associated with Paget disease of bone and frontotemporal dementia is caused by mutant valosin-containing protein. Nat Genet.

[21] Portsteffen H, Beyer A, Becker E, Epplen C, Pawlak A, Kunau WH, Dodt G (1997). Human PEX1 is mutated in complementation group 1 of the peroxisome biogenesis disorders. Nat Genet.

[22] Reuber BE, Germain-Lee E, Collins CS, Morrell JC, Ameritunga R, Moser HW, Valle D, Gould SJ (1997). Mutations in PEX1 are the most common cause of peroxisome biogenesis disorders. Nat Genet.

[23] Ozelius LJ, Hewett JW, Page CE, Bressman SB, Kramer PL, Shalish C, de Leon D, Brin MF, Raymond D, Corey DP (1997). The early-onset torsion dystonia gene (DYT1) encodes an ATP-binding protein. Nat Genet.

[24] Hazan J, Fonknechten N, Mavel D, Paternotte C, Samson D, Artiguenave F, Davoine CS, Cruaud C, Durr A, Wincker P (1999). Spastin, a new AAA protein, is altered in the most frequent form of autosomal dominant spastic paraplegia. Nat Genet.

[25] Casari G, De Fusco M, Ciarmatori S, Zeviani M, Mora M, Fernandez P, De Michele G, Filla A, Cocozza S, Marconi R (1998). Spastic paraplegia and OXPHOS impairment caused by mutations in paraplegin, a nuclear-encoded mitochondrial metalloprotease. Cell.

