# Supplementary material for: SPATA5 mutations cause a distinct autosomal recessive phenotype of intellectual disability, hypotonia and hearing loss
Source: Orphanet J Rare Dis. 2016 Sep 29;11:130. doi: 10.1186/s13023-016-0509-9 (PMC5041579; doi:10.1186/s13023-016-0509-9)
Supplement: Additional file 1: Table S1. — Comparison of phenotypes of individuals with mutations in SPATA5. (DOCX 15 kb) [file 13023_2016_509_MOESM1_ESM.docx]

**Additional file**

**Additional file 1: Table S1**

|  | individuals of family MR003 | individual B1 | published by Tanaka et al., 2015 | published by Kurata et al., 2016 |
| --- | --- | --- | --- | --- |
| Identified mutation | c.1822_1824del; p.Asp608del | c.[2081G>A];[989_991delCAA]; p.[Gly694Glu];[.Thr330del] | several | several |
| Intellectual disability | +++ | +++ | + | + |
| Contact avoidance | + | + | - | +/- |
| Microcephaly | + | borderline | + | + |
| Seizures | - | + | + | - |
| Motor development delay | + | + | + | + |
| Muscular hypotonia | +/- | + | + | + |
| Spasticity | - | - | +/- | + |
| Dystonia | - | + | - | +/- |
| Hearing impairment | +/- | + | + | + |
| Vision impairment | +/- | + | + | + |
| Gastrointestinal issues | + | - | + | - |
| Brain MRI | brain atrophy/normal | normal | various abnormalities | Cerebral atrohy, delayed myelination |
| EEG | abnormal | abnormal | abnormal | abnormal |

**+ = present, +++ = severe, - = not present**
